# Wilson’s Disease in a 17-Year-Old Male With Sickle Cell Trait: A Report of a Rare Case

**DOI:** 10.7759/cureus.51200

**Published:** 2023-12-27

**Authors:** Aditya Jain, Revat J Meshram, Sham Lohiya, Dinesh V Hinge, Shailesh Wandile

**Affiliations:** 1 Pediatrics, Jawaharlal Nehru Medical College, Datta Meghe Institute of Higher Education and Research, Wardha, IND

**Keywords:** multidisciplinary approach, vitamin-a deficiency, acalculous cholecystitis, adolescents, sickle cell trait, wilson's disease

## Abstract

This case report describes the atypical presentation of Wilson's disease in a 17-year-old male with sickle cell trait AS pattern. The patient initially presented with fever, generalized weakness, and joint pain, leading to an inconclusive diagnosis and unsuccessful initial treatment. A comprehensive re-evaluation revealed vitamin-A deficiency, adenoid hypertrophy, splenomegaly, and acalculous cholecystitis. Elevated copper levels in the 24-hour urine test confirmed the diagnosis of Wilson's disease. Treatment was modified to include amikacin, prednisolone, and Zinconia®, with analgesics for joint pain management. This case emphasizes the need for a thorough diagnostic approach and consideration of overlapping conditions in complex presentations, contributing to improved patient outcomes.

## Introduction

Sickle cell trait (SCT) is a genetic condition characterized by the presence of one abnormal hemoglobin gene (HbS) and one normal hemoglobin gene (HbA) in an individual's genetic makeup [1]. While SCT is generally considered a benign carrier state, it has been associated with various complications, including nephrotic syndrome and an increased susceptibility to certain infections [2]. Wilson's disease (WD), on the other hand, is a rare autosomal recessive disorder caused by mutations in the ATP7B gene, leading to impaired hepatic copper transport and subsequent copper accumulation in various organs, particularly the liver, brain, and cornea [3]. WD can present with a broad spectrum of symptoms, ranging from hepatic manifestations to neuropsychiatric disturbances, and its diagnosis often requires a high index of clinical suspicion and comprehensive investigations [4].

The coexistence of SCT and WD in the same patient represents a unique and challenging clinical scenario. While SCT is prevalent in populations with African ancestry, WD is a disorder with a global distribution with varying geographic and ethnic prevalence. The overlap of these two conditions poses diagnostic challenges, as both may independently contribute to a diverse array of clinical manifestations [5]. The association between SCT and nephrotic syndrome has been documented in the literature [6]. However, the simultaneous occurrence of SCT and WD in a pediatric patient with a history of nephrotic syndrome has not been extensively reported [7].

This case underscores the need for a holistic approach to patient care, considering the diverse genetic and environmental factors that may contribute to the clinical presentation. Additionally, it highlights the importance of re-evaluating cases with persistent symptoms despite initial treatment, especially in individuals with underlying genetic susceptibilities. Understanding the intricate interplay between SCT and WD is crucial for clinicians to navigate diagnostic challenges and implement appropriate therapeutic strategies [8]. This case report aims to contribute to the existing body of literature on the coexistence of these two conditions, providing valuable insights for clinicians involved in caring for patients with complex genetic profiles.

## Case presentation

A 17-year-old male with SCT AS pattern presented to the outpatient department with a one-month history of fever, generalized weakness, giddiness, and disturbed sleep. The patient had a previous diagnosis of SCT and nephrotic syndrome at the age of 14, with a history of hospitalisation for thrombocytopenia.

**Figure 1:**
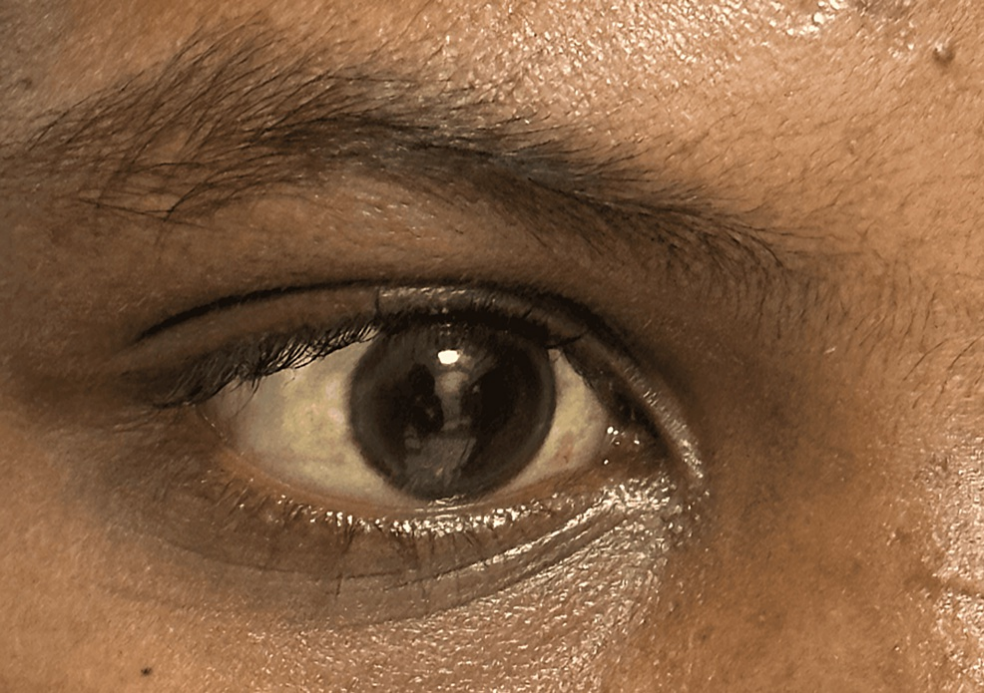
The figure shows yellowish discolouration of the eyes.

**Figure 2:**
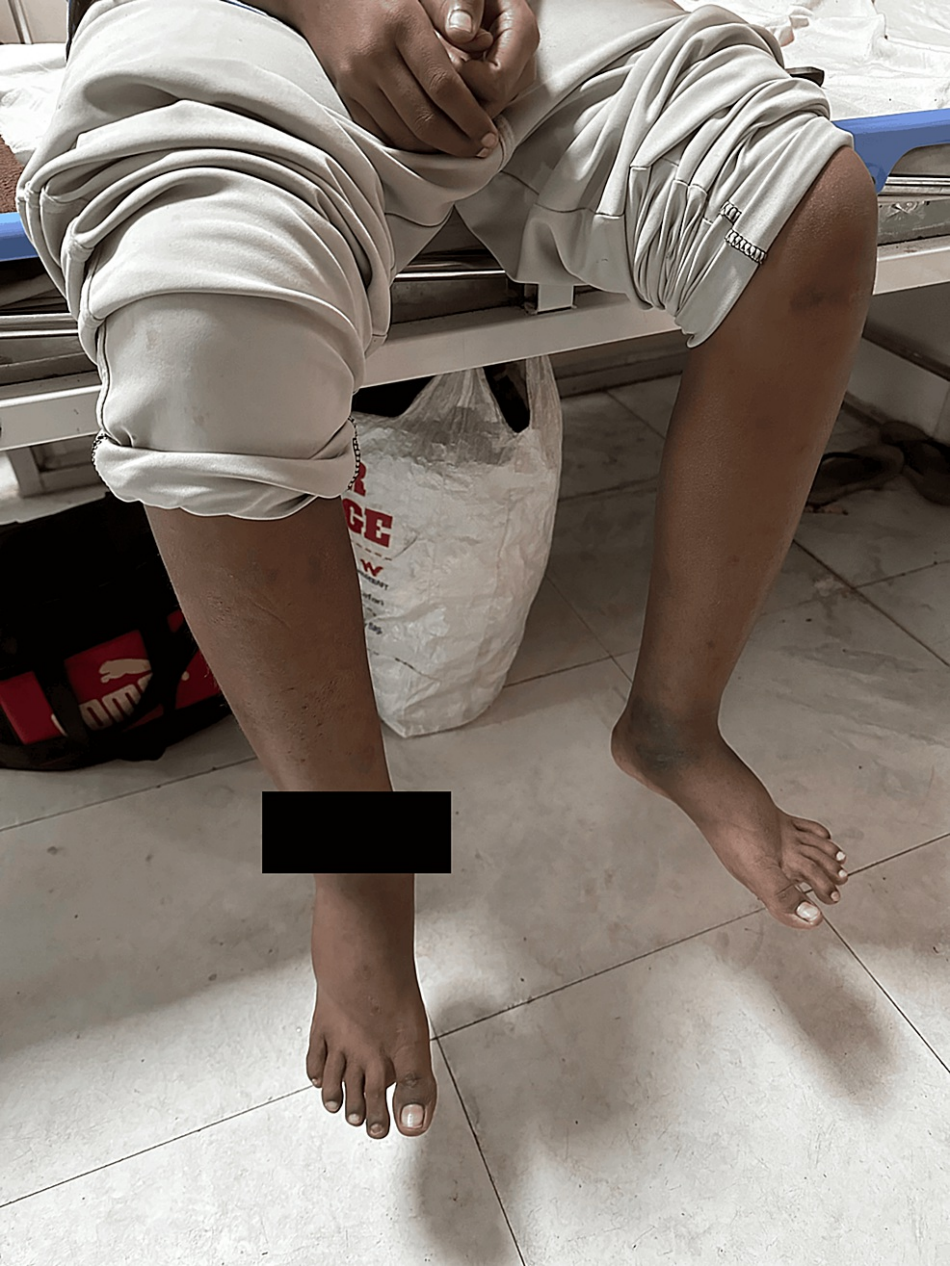
The figure shows pitting oedema over the lower limbs.

On examination, the patient exhibited yellowish discolouration of the eyes (Figure 1), pallor, and pitting oedema over the lower limbs (Figure 2). The paediatrician recommended admission for further evaluation. Blood tests were performed, including complete blood count, kidney function test, liver function test, and direct Coombs test (DCT). The DCT was positive. Treatment with antibiotics, analgesics, antipyretics, folic acid, and hydroxyurea was initiated, but the fever and joint pain persisted.

Additional assessments were initiated, given the absence of improvement in the patient's condition. Ophthalmology consultation was sought to investigate the persistent symptoms, and the examination revealed findings consistent with vitamin-A deficiency. Specifically, ocular manifestations indicative of vitamin-A deficiency were identified during the ophthalmologic assessment. Furthermore, nasal endoscopy conducted under local anaesthesia identified grade-I adenoid hypertrophy as part of the comprehensive evaluation. Simultaneously, abdominal ultrasound revealed significant splenomegaly (16 cm) accompanied by mild ascites and acalculous cholecystitis. Repeat blood tests were performed to delve deeper into the diagnostic process. Given the clinical presentation and in pursuit of a comprehensive evaluation, a copper 24-hour urine test was conducted. The results indicated a copper excretion of 17.85 ug/24 hours, providing crucial evidence for the diagnosis of WD. Combining these diagnostic findings supported a more accurate understanding of the patient's complex clinical condition. The treatment plan was modified based on the WD diagnosis. The patient was started on antibiotics (amikacin), prednisolone, and Zinconia®. Analgesics were administered for symptomatic relief of joint pain. Following the modified treatment regimen, the patient improved symptoms, including fever and joint pain resolution. Regular follow-up and monitoring were initiated to assess treatment response and manage potential complications.

## Discussion

SCT, characterized by the presence of one abnormal haemoglobin gene (HbS) and one normal haemoglobin gene (HbA), is generally considered a carrier state without significant clinical manifestations [2]. However, the association between SCT and nephrotic syndrome has been documented [6]. This case adds a layer of complexity by revealing a coexistence of SCT and WD, a rare autosomal recessive disorder characterized by impaired copper transport. Diagnosing WD in the presence of SCT can be challenging due to the diversity of symptoms shared by both conditions. The initial presentation with fever, weakness, and joint pain led to a broad range of differential diagnoses, including infectious etiologies and sickle cell-related complications [9]. The positive DCT prompted initial treatment for a presumed haematological disorder, emphasizing the importance of revisiting the diagnosis in cases of persistent symptoms [10].

The comprehensive re-evaluation of the patient involved collaboration with various specialists, including ophthalmologists, otolaryngologists, and radiologists. Thorough investigations identified vitamin-A deficiency, adenoid hypertrophy, splenomegaly, mild ascites, and acalculous cholecystitis, contributing to the eventual diagnosis of WD. This underscores the significance of a multidisciplinary approach in complex cases to uncover the full spectrum of clinical manifestations. The coexistence of SCT and WD, in this case, prompts intriguing questions about the interplay between genetic factors and environmental influences. SCT, more prevalent in populations with African ancestry [2], contrasts with WD, which exhibits a broader global distribution [3]. The simultaneous occurrence of these conditions in an individual emphasizes the importance of considering diverse genetic backgrounds in clinical practice.

Regarding the modification of the treatment plan prompted by the diagnosis of WD, antibiotics were introduced, specifically amikacin. The rationale behind this antibiotic choice and its role in the treatment plan should be elaborated upon in the context of its effectiveness against the underlying pathophysiology of WD. Additionally, prednisolone, a corticosteroid, was included in the treatment regimen. The mechanism of action of prednisolone in the context of WD and its contribution to the overall treatment strategy should be explained. Zinconia, a zinc supplement, was incorporated into the treatment plan. The specific role of zinc supplementation in managing WD and how it contributes to the overall therapeutic approach should be clarified. Symptomatic management of joint pain was initiated using analgesics. The choice of analgesics and their efficacy in alleviating joint pain in the context of WD should be detailed. Regular follow-ups were scheduled to monitor treatment response and adjust therapeutic interventions as needed. Still, the specific parameters monitored and the criteria for adjusting the treatment plan should be specified for clarity and completeness.

## Conclusions

The presented case of a 17-year-old male with SCT and the subsequent diagnosis of WD underscores the intricacies of overlapping genetic conditions and the challenges in reaching a definitive diagnosis. The coexistence of SCT and WD, with a history of nephrotic syndrome, accentuates the complexity of clinical presentations. The diagnostic journey involved collaboration among various specialists and highlighted the importance of a multidisciplinary approach to unravel the diverse manifestations of these genetic disorders. The case also raises intriguing questions about the interplay between genetic factors and geographic prevalence, emphasizing the need for broader considerations in clinical practice. The modified treatment plan for WD, incorporating antibiotics, prednisolone, and Zinconia, underscores the significance of tailoring therapeutic interventions based on a precise diagnosis. This case contributes valuable insights into the diagnostic challenges and management strategies for patients with overlapping genetic conditions, emphasizing the importance of ongoing research to enhance our understanding of such complex clinical scenarios.
